# Spontaneous transverse colon volvulus in a patient with Duchenne muscular dystrophy: An unreported complication

**DOI:** 10.1016/j.radcr.2022.12.062

**Published:** 2023-01-18

**Authors:** Pietro Pitrone, Antonino Cattafi, Francesca Magnani, Maria Ludovica Carerj, Italo Giuseppe Bellone, Giuseppe Nirta, Enrico Monsù, Dora Bonanno, Renato Trimarchi, Alessandro La Face, Maria Adele Marino, Carmelo Sofia

**Affiliations:** a Section of Radiological Sciences, Department of Biomedical Sciences and Morphological and Functional Imaging, University of Messina, Policlinico ``G. Martino'' Via Consolare Valeria 1, 98100, Messina, Italy; b Department of Radiodiagnostic, Oncologic Radiotherapy and Ematology, Università Cattolica del Sacro Cuore, Fondazione Policlinico Universitario A. Gemelli IRCCS, L.go A. F.Vito 1 Gemelli 8, 00168, Rome, Italy

**Keywords:** Duchenne muscular dystrophy, Gastrointestinal, Chronic constipation, Transverse colon volvulus

## Abstract

A 22-year-old male patient with Duchenne muscular dystrophy (DMD) and chronic constipation presents to the emergency room with severe abdominal pain and hive closed to feces and gas. Contrast-enhanced computed tomography of the abdomen demonstrates mechanical ileus due to volvulus of the transverse colon: torsion of the transverse mesocolon is confirmed and subtotaly colectomy is performed, revealing multiple ischemic areas with focal perforations. DMD is frequently associated with gastrointestinal motility disorders, including chronic constipation and life-threatening conditions like intestinal pseudo-obstruction and sigmoid volvulus. To date, transverse colic localization of volvolus represents an unreported condition among patients with DMD.

## Introduction

Duchenne muscular dystrophy (DMD) represents the most common inherited neuromuscular disorder in children. It is associated with mutations in the gene coding for dystrophin protein and leads to progressive muscular weakness and disability from a very young age [1], [2], [3], followed by a severe cardio-respiratory failure between the second and the third decade [4], [5], [6]. Other minor symptoms involve the urinary and, most importantly, the gastrointestinal tract, including motility disorders (gastric distension, chronic constipation, and diarrhea) as well as life-threatening conditions like intestinal pseudo-obstruction and volvulus, with reported sigmoid localization [6], [7], [8], [9], [10].

## Case report

**Fig. 1:**
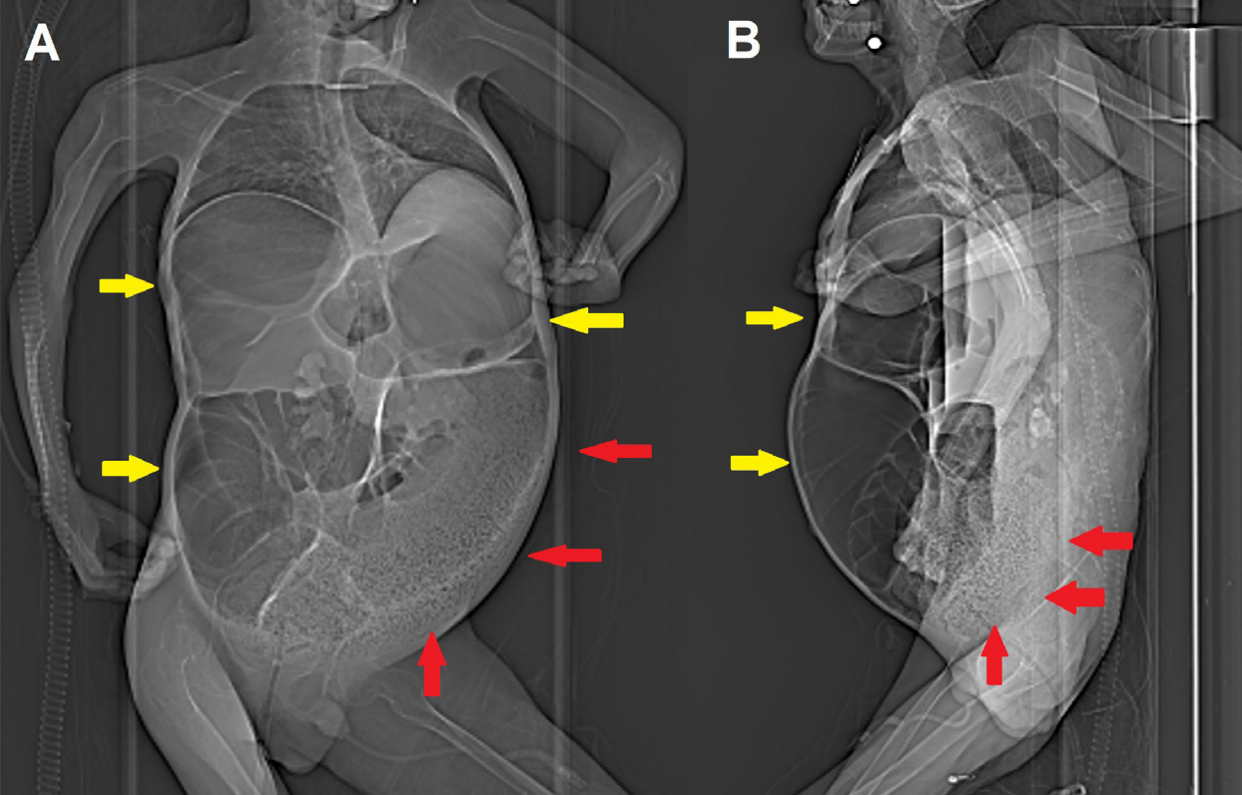
Scout images (anteroposterior [A] and lateral view [B]) obtained before contrast-enhanced CT already show extensive bowel dilatation (yellow arrows) and left colon fecaloma (red arrows).

**Fig. 2:**
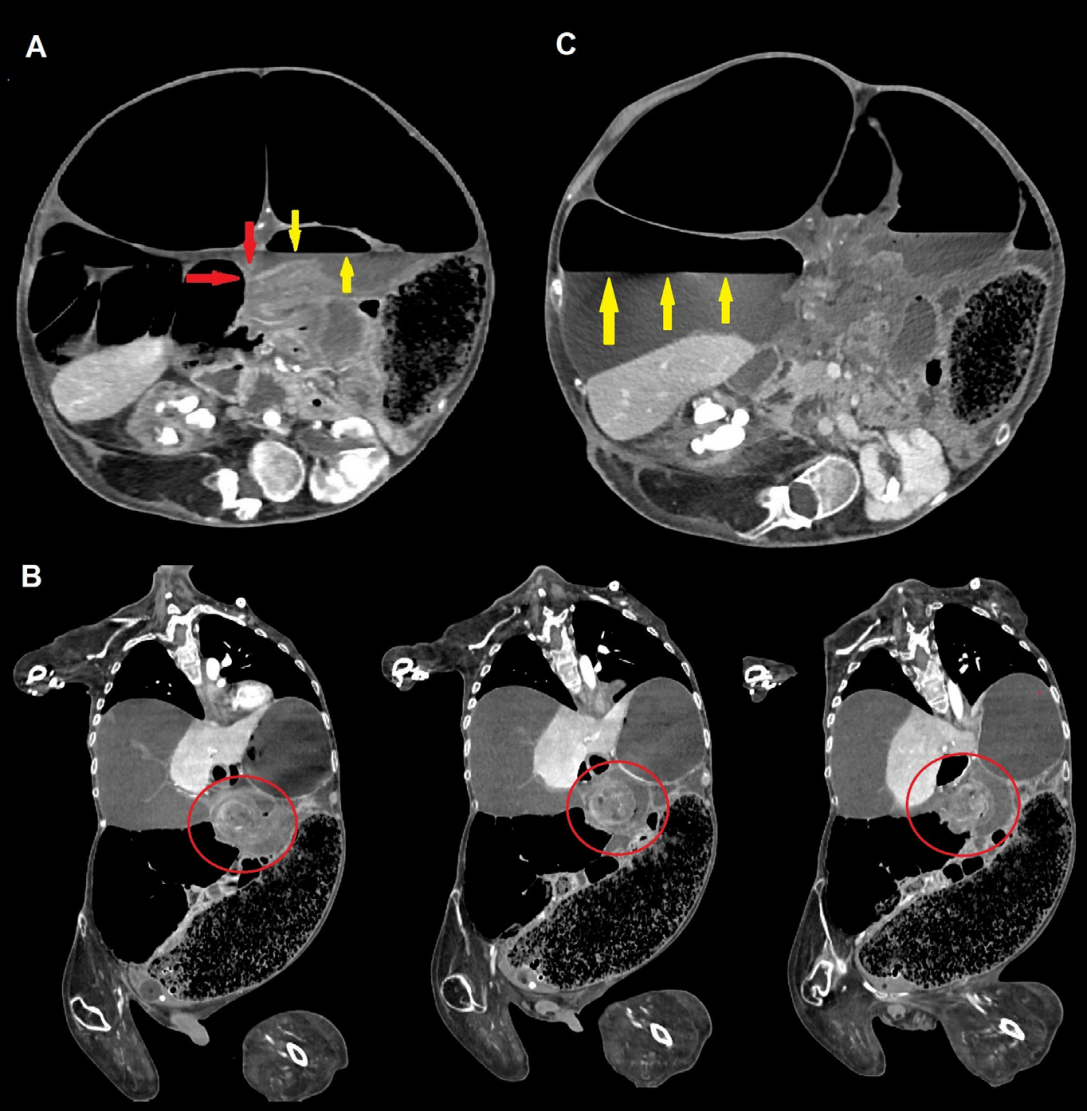
Contrast-enhanced CT scans of the abdomen demonstrating volvulus of the transverse colon. Axial scans (A) show an abrupt caliber change (“*beak sign*” [red arrows], common in many types of mechanical ileum) representing the site of occlusion, associated with proximal air-fluid level (yellow arrows). Coronal view (B), obtained with multiplanar reconstructions (MPR), demonstrates the same loop twisting around the long axis of its meso (“*whirl sign*” [red circles]); most of the right colon is displaced, as well as jejunal loops (medially), due to partial involvement of the root of the mesentery. Significant right colic dilatation (with atony) is present, with a caliber of caecum measuring more than 17 cm and an evident air-fluid level (C [yellow arrows]).

**Fig. 3:**
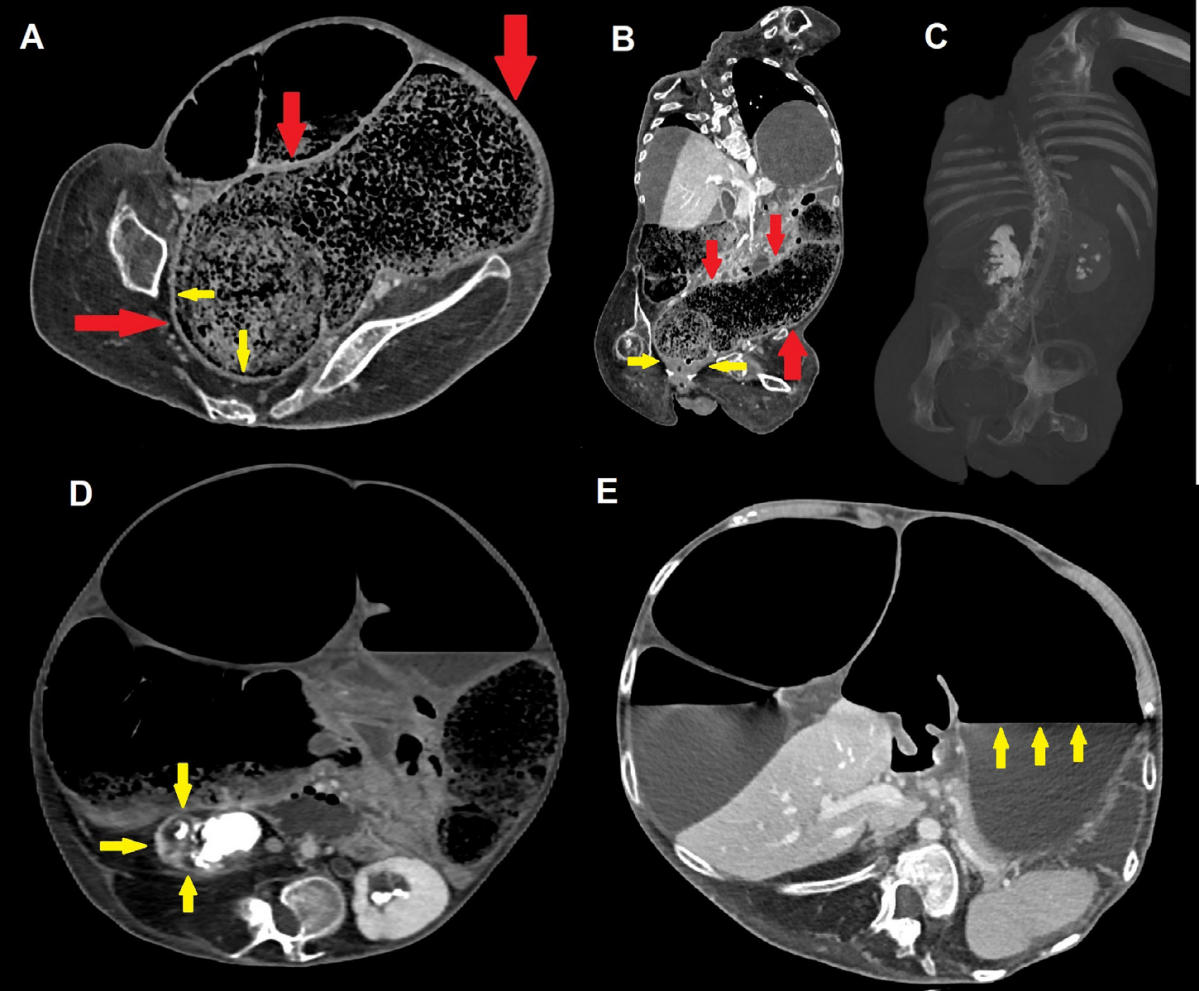
Contrast-enhanced CT scans on a lower level (A) and coronal view on MPR (B) demonstrate an extensive recto-sigmoid fecaloma (measuring more than 30 × 10 cm; red arrows); chronic thickening of rectal walls is visible (yellow arrows), as well as compressive bilateral I-grade hydronephrosis, renal stones and thinning of renal parenchyma (frequent in DMD; C and D [yellow arrows]). Dilatation with atonia and an evident fluid-air level also involves the stomach (E [yellow arrows]), further witnessing the multifocal nature of GI motility disturbances in patients with DMD.

**Fig. 4:**
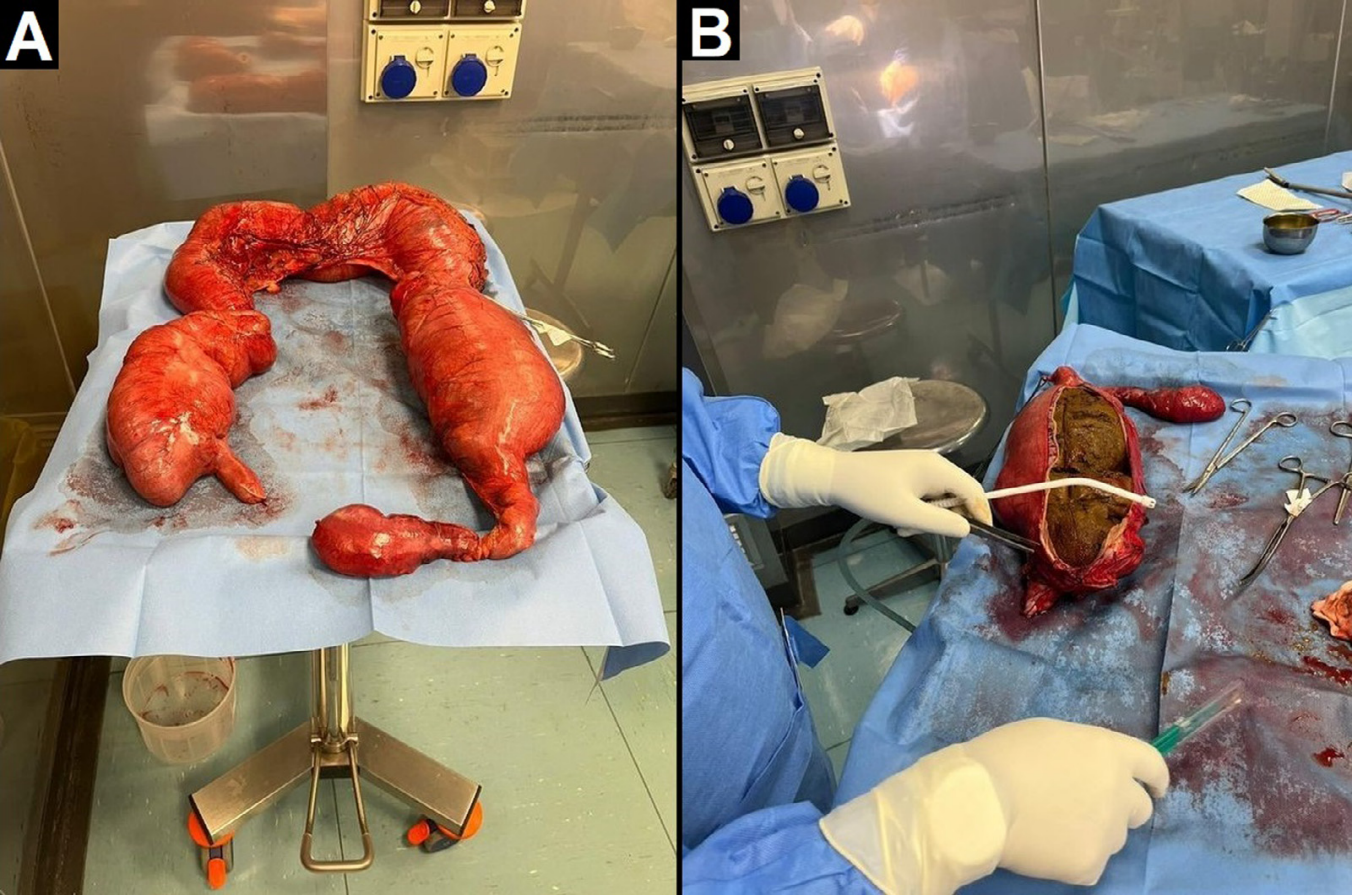
Subtotal colectomy. Multiple ischemic areas are visible, especially at the level of the transverse colon, where focal perforation with initial fecal leakage is present. There is also distension of the caecum and descending colon (A), the latter due to the known recto-sigmoid fecaloma (B).

A 22-year-old male patient with DMD, cardio-pulmonary (requiring noninvasive ventilation) and renal failure, and a history of constipation (managed with Movicol and other pro-kinetics) presents to the emergency room with severe nausea and abdominal pain for the last 2 days. The clinical evaluation shows a rounded and tender abdomen associated with a hive closed to feces and gas. Contrast-enhanced computed tomography of the abdomen (scout images in Fig. 1) demonstrates volvulus of the transverse colon associated with mechanical ileus (Fig. 2); the left colon is collapsed and followed by an extensive recto-sigmoid fecaloma (Fig. 3). The patient has thus demanded emergency surgery: diffuse megacolon with atony is seen and multiple ischemic areas are revealed, especially within the transverse segment where focal perforation with initial fecal leakage is present. At this level, complete torsion of the transverse mesocolon is confirmed: thus, subtotal colectomy is performed (Fig. 4).

## Discussion

DMD represents the most frequent inherited muscular dystrophy (affecting nearly one in 3300 male births) and the most common genetic neuromuscular disorder in children. It is caused by recessive mutations in the gene (located at Xp21) which codes for the dystrophin protein and leads to progressive striated muscular weakness and severe physical disability (with loss of ambulation around the age of 12 years) [1], [2], [3].

With time, spinal and chest wall deformities along with impairment of respiratory muscle function lead to hypercapnic respiratory failure, whereas cardiac muscle involvement determines congestive heart failure, with expected death around the second or third decade [4], [5], [6].

In addition, most patients experience gastrointestinal and, to a lesser extent, urinary symptoms (ie, urinary incontinence, hesitancy, straining, weak stream, intermittency nephrolithiasis, and renal insufficiency) already from a young age. Dysphagia (due to swallowing impairment), gastroesophageal reflux, collection of gastric air, chronic constipation (in up to 46,7% of patients) and diarrhea (with possible alternating pattern), blood in stool and fecal incontinence may occur [7], [8], [9], as well as life-threatening complications like acute gastric dilatation, gastroparesis, and intestinal pseudo-obstruction, the latter with dilated and fluid-filled small intestine and colon and possible acute respiratory failure. For this reason, young adults with DMD and a history of abdominal bloating should be routinely investigated with abdominal radiography [10].

Autopsy studies have demonstrated edema, fatty infiltration, fragmentation, fibrosis, and waxy degeneration of smooth muscle, resulting in atrophy and thinning of the bowel wall [11,12]. Furthermore, alterations of the myenteric plexus (with reduced myoelectrical slow wave activity) and reduced availability of nitric oxide (due to lack of dystrophin, which acts as an anchor for No-synthase) have been advocated, resulting in impaired gastro-intestinal motility [13], [14], [15], [16].

Another rare GI complication is volvulus, with reported sigmoid localization in patients with a long-standing history of abdominal bloating and constipation along with episodes of pseudo-obstruction and severe bowel wall alterations [10].

However, no experience in literature is reported about transverse volvulus.

## Conclusion

DMD is associated with many gastrointestinal symptoms, including motility disturbances (mostly chronic constipation) and acute complications like pseudo-obstruction and sigmoid volvulus. No mention is made of transverse colic localization in literature; thus, such a possibility must always be evaluated.

## Ethical statement

All procedures performed in the study were in accordance with the ethical standards of the institutional and/or national research committee and with the 1964 Helsinki declaration and its later amendments or comparable ethical standards.

## Consent to participate

Not applicable for this type of the work (case report).

## Patient consent

The patient provided a written informed consent for using anonymized data for publication.
